# *rac*-Methyl 3-(2-meth­oxy­phen­yl)-3a,4-di­hydro-3*H*-chromeno[4,3-*c*]isoxazole-3a-carboxyl­ate

**DOI:** 10.1107/S1600536813008635

**Published:** 2013-04-05

**Authors:** S. Paramasivam, J. Srinivasan, P. R. Seshadri, M. Bakthadoss

**Affiliations:** a Post Graduate and Research Department of Physics, Agurchand Manmull Jain College, Chennai 600 114, India; b Department of Organic Chemistry, University of Madras, Guindy Campus, Chennai 600 025, India

## Abstract

The title compound, C_19_H_17_NO_5_, comprising two stereogenic C atoms of the same configuration, crystallizes in a centrosymmetric space group as a racemate. The pyran ring adopts a half-chair conformation, while the isoxazole ring adopts an envelope conformation with the C atom bonded to the meth­oxy­phenyl group as the flap. The dihedral angle between the mean plane of the pyran ring and the adjacent benzene ring is 5.86 (5)°. In the crystal, mol­ecules are linked by a weak C—H⋯O hydrogen bond, forming a chain along the *a* axis.

## Related literature

For the biological activity of isoxazole and benzpyran derivatives, see: Winn *et al.* (1976 ▶); Rozman *et al.* (2002 ▶); Caine (1993 ▶). For conformational analysis and puckering parameters, see: Cremer & Pople, (1975 ▶). For a related structure, see: Paramasivam *et al.* (2012 ▶).
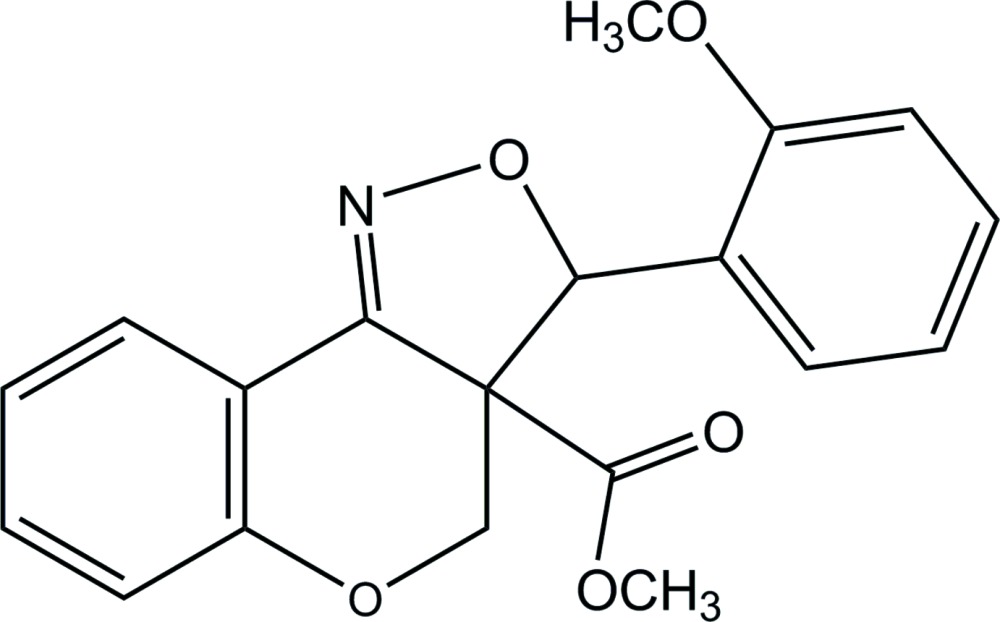


## Experimental

### 

#### Crystal data

C_19_H_17_NO_5_
*M* *_r_* = 339.34
Triclinic, 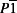

*a* = 9.4804 (4) Å
*b* = 9.6401 (4) Å
*c* = 10.7013 (5) Å
α = 81.308 (2)°
β = 67.801 (2)°
γ = 69.085 (2)°
*V* = 845.71 (6) Å^3^
*Z* = 2
Mo *K*α radiation
μ = 0.10 mm^−1^
*T* = 298 K
0.30 × 0.25 × 0.20 mm

#### Data collection

Bruker SMART APEXII area-detector diffractometer
Absorption correction: multi-scan (*SADABS*; Bruker, 2008 ▶) *T*_min_ = 0.971, *T*_max_ = 0.981
12631 measured reflections
3462 independent reflections
3012 reflections with *I* > 2σ(*I*)
*R*_int_ = 0.030

#### Refinement

*R*[*F* ^2^ > 2σ(*F* ^2^)] = 0.040
*wR*(*F* ^2^) = 0.112
*S* = 1.04
3462 reflections
229 parameters
H-atom parameters constrained
Δρ_max_ = 0.22 e Å^−3^
Δρ_min_ = −0.19 e Å^−3^

### 

Data collection: *APEX2* (Bruker, 2008 ▶); cell refinement: *SAINT* (Bruker, 2008 ▶); data reduction: *SAINT*; program(s) used to solve structure: *SHELXS97* (Sheldrick, 2008 ▶); program(s) used to refine structure: *SHELXL97* (Sheldrick, 2008 ▶); molecular graphics: *ORTEP-3 for Windows* (Farrugia, 2012 ▶) and *PLATON* (Spek, 2009 ▶); software used to prepare material for publication: *SHELXL97*, *PLATON* and *publCIF* (Westrip, 2010 ▶).

## Supplementary Material

Crystal structure: contains datablock(s) I, global. DOI: 10.1107/S1600536813008635/is5259sup1.cif

Structure factors: contains datablock(s) I. DOI: 10.1107/S1600536813008635/is5259Isup2.hkl

Supplementary material file. DOI: 10.1107/S1600536813008635/is5259Isup3.cml

Additional supplementary materials:  crystallographic information; 3D view; checkCIF report

## Figures and Tables

**Table 1:** Hydrogen-bond geometry (Å, °)

*D*—H⋯*A*	*D*—H	H⋯*A*	*D*⋯*A*	*D*—H⋯*A*
C17—H17⋯O3^i^	0.93	2.42	3.3084 (19)	159

Symmetry code: (i) 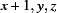.

**Fig. 1.**
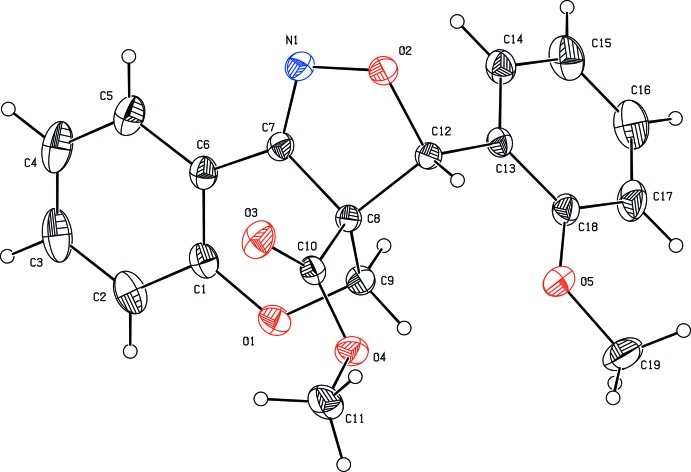
The molecular structure of the title compound, showing the atom-numbering scheme and displacement ellipsoids drawn at the 20% probability level.

## supplementary crystallographic information

### Comment

As a continuation of our research related to isoxazole containing chromenoisoxazole moiety, we analyzed the crystal structure of *rac-*methyl 3-(2-methoxyphenyl)-1-phenyl-3,3a,4,9b- tetrahydro-1*H*-chromeno[4,3-*c*]isoxazole-3a-carboxylate (Paramasivam *et al.*, 2012). The present compound exhibits the pronounced similarity to the previous ones, either in bond lengths and angles as well as in molecular conformation. Isoxazole derivative is used for the treatment of rheumatoid arthritis (Rozman *et al.*, 2002) whereas benzopyran derivatives exhibit anti-depressant activities (Winn *et al.*, 1976) and in the treatment of impulsive-disorder disease (Caine, 1993). On this grounds, the title compound was chosen for X-ray structure analysis (Fig. 1).

The pyran ring (O1/C1/C6–C9) adopts a half-chair conformation with the puckering parameters (Cremer & Pople, 1975) being q_2_ = 0.395 (1) Å, q_3_ = -0.296 (1) Å, Q_T_ = 0.494 (1) Å and the five-membered ring isoxazole (O2/N1/C7/C8/C12) adopts an envelope conformation with atom C12 as the flap, with puckering parameters being q_2_ = 0.246 (1) Å and Φ_2_ = 143.9 (3)°. The dihedral angle between the pyran and the benzene ring (C1–C6) is 5.86 (5)°. Also the dihedral angle between the chromeno ring (fusion of benzene and pyran rings) and isoxazole ring is 11.87 (5)°. In the chromenoisoxazole moiety, the dihedral angle between the benzene and isoxazole ring is 7.71 (6)°. The dihedral angle between the pyran and isoxazole ring is 13.40 (5)°. The geometric parameters of the title compound agree well with the reported similar structure (Paramasivam *et al.*, 2012). The crystal packing is stabilized by C—H···O hydrogen bonds (Table 1).

### Experimental

A solution of (*E*)-methyl 2-({2-[(*E*)-(hydroxyimino)methyl]phenoxy}methyl)-3-(2-methoxyphenyl)acrylate (2 mmol) in CCl_4_ at 0–10 °C was added pinch wise NCS (4 mmol) over 3 h. After Et_3_N (4 mmol) was added the reaction mixture was stirred at room temperature for 2 h. After completion of the reaction, reaction mixture was evaporated under reduced pressure and the resulting crude mass was diluted with water (15 ml) and extracted with ethyl acetate (3 × 15 ml). The combining organic layer was washed with brine (2 × 10 ml) and dried over anhydrous Na_2_SO_4_. The organic layer was evaporated and purified by column chromatography (silica gel 60–120 mesh 7% EtOAc in hexanes) to provide the desired pure product methyl 3-(2-methoxyphenyl)-3a,4-dihydro-3*H*-chromeno[4,3-*c*]isoxazole-3a-carboxylate as a colorless solid.

### Refinement

Hydrogen atoms were positioned geometrically and allowed to ride on their parent atoms, with C—H = 0.93–0.97 Å and with *U*_iso_(H) = 1.5*U*_eq_(C) for methyl H atoms and 1.2*U*_eq_(C) for other H atoms.

### Crystal data

**Table:**

C_19_H_17_NO_5_	*Z* = 2
*M**_r_* = 339.34	*F*(000) = 356
Triclinic, *P*1	Triclinic
Hall symbol: -P 1	*D*_x_ = 1.333 Mg m^−^^3^
*a* = 9.4804 (4) Å	Mo *K*α radiation, λ = 0.71073 Å
*b* = 9.6401 (4) Å	Cell parameters from 3462 reflections
*c* = 10.7013 (5) Å	θ = 2.1–26.4°
α = 81.308 (2)°	µ = 0.10 mm^−^^1^
β = 67.801 (2)°	*T* = 298 K
γ = 69.085 (2)°	Block, colourless
*V* = 845.71 (6) Å^3^	0.30 × 0.25 × 0.20 mm

### Data collection

**Table:**

Bruker SMART APEXII area-detector diffractometer	3462 independent reflections
Radiation source: fine-focus sealed tube	3012 reflections with *I* > 2σ(*I*)
Graphite monochromator	*R*_int_ = 0.030
ω and φ scans	θ_max_ = 26.4°, θ_min_ = 2.1°
Absorption correction: multi-scan (*SADABS*; Bruker, 2008)	*h* = −11→11
*T*_min_ = 0.971, *T*_max_ = 0.981	*k* = −11→12
12631 measured reflections	*l* = −13→13

### Refinement

**Table:**

Refinement on *F*^2^	Secondary atom site location: difference Fourier map
Least-squares matrix: full	Hydrogen site location: inferred from neighbouring sites
*R*[*F*^2^ > 2σ(*F*^2^)] = 0.040	H-atom parameters constrained
*wR*(*F*^2^) = 0.112	*w* = 1/[σ^2^(*F*_o_^2^) + (0.0548*P*)^2^ + 0.182*P*] where *P* = (*F*_o_^2^ + 2*F*_c_^2^)/3
*S* = 1.04	(Δ/σ)_max_ < 0.001
3462 reflections	Δρ_max_ = 0.22 e Å^−^^3^
229 parameters	Δρ_min_ = −0.19 e Å^−^^3^
0 restraints	Extinction correction: *SHELXL97* (Sheldrick, 2008), Fc^*^=kFc[1+0.001xFc^2^λ^3^/sin(2θ)]^-1/4^
Primary atom site location: structure-invariant direct methods	Extinction coefficient: 0.093 (6)

### Special details

**Table:**

Geometry. All e.s.d.'s (except the e.s.d. in the dihedral angle between two l.s. planes) are estimated using the full covariance matrix. The cell e.s.d.'s are taken into account individually in the estimation of e.s.d.'s in distances, angles and torsion angles; correlations between e.s.d.'s in cell parameters are only used when they are defined by crystal symmetry. An approximate (isotropic) treatment of cell e.s.d.'s is used for estimating e.s.d.'s involving l.s. planes.
Refinement. Refinement of *F*^2^ against ALL reflections. The weighted *R*-factor *wR* and goodness of fit *S* are based on *F*^2^, conventional *R*-factors *R* are based on *F*, with *F* set to zero for negative *F*^2^. The threshold expression of *F*^2^ > σ(*F*^2^) is used only for calculating *R*-factors(gt) *etc*. and is not relevant to the choice of reflections for refinement. *R*-factors based on *F*^2^ are statistically about twice as large as those based on *F*, and *R*- factors based on ALL data will be even larger.

### Fractional atomic coordinates and isotropic or equivalent isotropic displacement parameters (Å^2^)

**Table:**

	*x*	*y*	*z*	*U*_iso_*/*U*_eq_	
C1	0.76808 (16)	0.07314 (16)	0.63411 (13)	0.0495 (3)	
C2	0.7901 (2)	−0.0399 (2)	0.55433 (17)	0.0684 (5)	
H2	0.8490	−0.1376	0.5680	0.082*	
C3	0.7231 (2)	−0.0043 (3)	0.4548 (2)	0.0868 (7)	
H3	0.7355	−0.0791	0.4021	0.104*	
C4	0.6378 (3)	0.1410 (3)	0.4322 (2)	0.0881 (6)	
H4	0.5961	0.1633	0.3630	0.106*	
C5	0.6143 (2)	0.2526 (2)	0.51121 (16)	0.0660 (4)	
H5	0.5564	0.3500	0.4957	0.079*	
C6	0.67764 (16)	0.21950 (16)	0.61527 (13)	0.0471 (3)	
C7	0.65159 (14)	0.32946 (14)	0.70800 (12)	0.0408 (3)	
C8	0.70310 (13)	0.27662 (13)	0.82869 (12)	0.0364 (3)	
C9	0.86085 (15)	0.14797 (14)	0.78248 (14)	0.0441 (3)	
H9A	0.9423	0.1821	0.7119	0.053*	
H9B	0.8986	0.1103	0.8575	0.053*	
C10	0.57037 (15)	0.22930 (13)	0.94184 (12)	0.0402 (3)	
C11	0.5050 (2)	0.09585 (19)	1.14593 (15)	0.0660 (4)	
H11A	0.4436	0.1773	1.2068	0.099*	
H11B	0.5573	0.0116	1.1917	0.099*	
H11C	0.4343	0.0707	1.1153	0.099*	
C12	0.70663 (14)	0.42463 (13)	0.86467 (12)	0.0385 (3)	
H12	0.6616	0.4354	0.9629	0.046*	
C13	0.87197 (14)	0.44101 (14)	0.81075 (13)	0.0410 (3)	
C14	0.92427 (19)	0.51937 (18)	0.69297 (15)	0.0559 (4)	
H14	0.8557	0.5657	0.6447	0.067*	
C15	1.0783 (2)	0.5294 (2)	0.64626 (18)	0.0781 (5)	
H15	1.1132	0.5817	0.5667	0.094*	
C16	1.1786 (2)	0.4619 (3)	0.7180 (2)	0.0845 (6)	
H16	1.2815	0.4692	0.6866	0.101*	
C17	1.12966 (19)	0.3833 (2)	0.8361 (2)	0.0708 (5)	
H17	1.1990	0.3378	0.8837	0.085*	
C18	0.97535 (16)	0.37288 (15)	0.88296 (15)	0.0493 (3)	
C19	1.0138 (3)	0.2223 (2)	1.0745 (2)	0.0877 (6)	
H19A	1.1042	0.1442	1.0219	0.132*	
H19B	0.9531	0.1807	1.1551	0.132*	
H19C	1.0517	0.2909	1.0983	0.132*	
N1	0.59522 (14)	0.47046 (13)	0.69528 (12)	0.0500 (3)	
O1	0.83574 (12)	0.03193 (10)	0.73258 (10)	0.0533 (3)	
O2	0.59926 (11)	0.53548 (10)	0.80421 (10)	0.0503 (3)	
O3	0.43272 (12)	0.27371 (14)	0.94996 (11)	0.0638 (3)	
O4	0.62574 (12)	0.13826 (11)	1.03084 (10)	0.0543 (3)	
O5	0.91320 (14)	0.29842 (12)	0.99727 (11)	0.0631 (3)	

### Atomic displacement parameters (Å^2^)

**Table:**

	*U*^11^	*U*^22^	*U*^33^	*U*^12^	*U*^13^	*U*^23^
C1	0.0439 (7)	0.0600 (8)	0.0443 (7)	−0.0261 (6)	−0.0036 (6)	−0.0086 (6)
C2	0.0591 (9)	0.0735 (10)	0.0691 (10)	−0.0301 (8)	−0.0017 (8)	−0.0262 (8)
C3	0.0716 (12)	0.1236 (18)	0.0755 (12)	−0.0442 (13)	−0.0075 (10)	−0.0495 (12)
C4	0.0765 (13)	0.137 (2)	0.0656 (11)	−0.0388 (13)	−0.0267 (10)	−0.0296 (12)
C5	0.0616 (10)	0.0929 (12)	0.0524 (8)	−0.0310 (9)	−0.0235 (7)	−0.0032 (8)
C6	0.0423 (7)	0.0615 (8)	0.0412 (6)	−0.0252 (6)	−0.0105 (5)	−0.0016 (6)
C7	0.0331 (6)	0.0483 (7)	0.0433 (6)	−0.0173 (5)	−0.0140 (5)	0.0041 (5)
C8	0.0310 (6)	0.0381 (6)	0.0404 (6)	−0.0124 (5)	−0.0122 (5)	0.0005 (5)
C9	0.0371 (6)	0.0408 (6)	0.0520 (7)	−0.0090 (5)	−0.0155 (5)	−0.0037 (5)
C10	0.0419 (7)	0.0419 (6)	0.0416 (6)	−0.0195 (5)	−0.0139 (5)	−0.0018 (5)
C11	0.0900 (12)	0.0707 (10)	0.0456 (8)	−0.0495 (9)	−0.0156 (8)	0.0110 (7)
C12	0.0333 (6)	0.0377 (6)	0.0448 (6)	−0.0116 (5)	−0.0146 (5)	0.0010 (5)
C13	0.0363 (6)	0.0436 (6)	0.0462 (6)	−0.0167 (5)	−0.0138 (5)	−0.0025 (5)
C14	0.0581 (9)	0.0685 (9)	0.0499 (7)	−0.0350 (7)	−0.0172 (7)	0.0043 (7)
C15	0.0699 (11)	0.1101 (15)	0.0617 (10)	−0.0595 (11)	−0.0034 (9)	−0.0006 (9)
C16	0.0441 (9)	0.1152 (16)	0.0962 (14)	−0.0423 (10)	−0.0056 (9)	−0.0179 (12)
C17	0.0447 (8)	0.0802 (11)	0.0975 (13)	−0.0169 (8)	−0.0346 (9)	−0.0135 (10)
C18	0.0436 (7)	0.0501 (7)	0.0605 (8)	−0.0152 (6)	−0.0242 (6)	−0.0034 (6)
C19	0.1082 (16)	0.0698 (11)	0.0944 (14)	−0.0083 (11)	−0.0694 (13)	0.0104 (10)
N1	0.0463 (6)	0.0503 (6)	0.0596 (7)	−0.0170 (5)	−0.0276 (5)	0.0080 (5)
O1	0.0579 (6)	0.0407 (5)	0.0587 (6)	−0.0131 (4)	−0.0190 (5)	−0.0054 (4)
O2	0.0451 (5)	0.0388 (5)	0.0712 (6)	−0.0086 (4)	−0.0302 (5)	0.0000 (4)
O3	0.0389 (5)	0.0922 (8)	0.0632 (6)	−0.0304 (5)	−0.0169 (5)	0.0098 (6)
O4	0.0582 (6)	0.0577 (6)	0.0492 (5)	−0.0273 (5)	−0.0190 (5)	0.0140 (4)
O5	0.0719 (7)	0.0639 (6)	0.0685 (7)	−0.0266 (6)	−0.0434 (6)	0.0168 (5)

### Geometric parameters (Å, º)

**Table:**

C1—O1	1.3718 (17)	C11—H11A	0.9600
C1—C2	1.394 (2)	C11—H11B	0.9600
C1—C6	1.395 (2)	C11—H11C	0.9600
C2—C3	1.379 (3)	C12—O2	1.4521 (15)
C2—H2	0.9300	C12—C13	1.5102 (16)
C3—C4	1.382 (3)	C12—H12	0.9800
C3—H3	0.9300	C13—C14	1.3829 (19)
C4—C5	1.372 (3)	C13—C18	1.3959 (18)
C4—H4	0.9300	C14—C15	1.388 (2)
C5—C6	1.402 (2)	C14—H14	0.9300
C5—H5	0.9300	C15—C16	1.370 (3)
C6—C7	1.4544 (19)	C15—H15	0.9300
C7—N1	1.2775 (17)	C16—C17	1.380 (3)
C7—C8	1.5053 (16)	C16—H16	0.9300
C8—C9	1.5237 (16)	C17—C18	1.392 (2)
C8—C10	1.5327 (16)	C17—H17	0.9300
C8—C12	1.5476 (16)	C18—O5	1.3604 (18)
C9—O1	1.4352 (16)	C19—O5	1.4296 (19)
C9—H9A	0.9700	C19—H19A	0.9600
C9—H9B	0.9700	C19—H19B	0.9600
C10—O3	1.1918 (15)	C19—H19C	0.9600
C10—O4	1.3225 (15)	N1—O2	1.4229 (15)
C11—O4	1.4531 (17)		
			
O1—C1—C2	116.64 (14)	O4—C11—H11C	109.5
O1—C1—C6	122.61 (12)	H11A—C11—H11C	109.5
C2—C1—C6	120.73 (15)	H11B—C11—H11C	109.5
C3—C2—C1	118.83 (18)	O2—C12—C13	110.92 (10)
C3—C2—H2	120.6	O2—C12—C8	102.91 (9)
C1—C2—H2	120.6	C13—C12—C8	114.29 (10)
C2—C3—C4	120.97 (17)	O2—C12—H12	109.5
C2—C3—H3	119.5	C13—C12—H12	109.5
C4—C3—H3	119.5	C8—C12—H12	109.5
C5—C4—C3	120.49 (18)	C14—C13—C18	119.30 (12)
C5—C4—H4	119.8	C14—C13—C12	122.56 (12)
C3—C4—H4	119.8	C18—C13—C12	118.14 (11)
C4—C5—C6	119.91 (19)	C13—C14—C15	120.48 (16)
C4—C5—H5	120.0	C13—C14—H14	119.8
C6—C5—H5	120.0	C15—C14—H14	119.8
C1—C6—C5	119.02 (14)	C16—C15—C14	119.62 (16)
C1—C6—C7	117.43 (12)	C16—C15—H15	120.2
C5—C6—C7	123.53 (14)	C14—C15—H15	120.2
N1—C7—C6	126.69 (12)	C15—C16—C17	121.24 (15)
N1—C7—C8	114.75 (11)	C15—C16—H16	119.4
C6—C7—C8	118.48 (11)	C17—C16—H16	119.4
C7—C8—C9	106.98 (10)	C16—C17—C18	119.23 (16)
C7—C8—C10	109.18 (9)	C16—C17—H17	120.4
C9—C8—C10	112.00 (10)	C18—C17—H17	120.4
C7—C8—C12	99.03 (9)	O5—C18—C17	124.89 (14)
C9—C8—C12	118.65 (10)	O5—C18—C13	114.98 (12)
C10—C8—C12	109.87 (9)	C17—C18—C13	120.13 (15)
O1—C9—C8	109.50 (10)	O5—C19—H19A	109.5
O1—C9—H9A	109.8	O5—C19—H19B	109.5
C8—C9—H9A	109.8	H19A—C19—H19B	109.5
O1—C9—H9B	109.8	O5—C19—H19C	109.5
C8—C9—H9B	109.8	H19A—C19—H19C	109.5
H9A—C9—H9B	108.2	H19B—C19—H19C	109.5
O3—C10—O4	124.43 (12)	C7—N1—O2	107.98 (10)
O3—C10—C8	123.42 (12)	C1—O1—C9	116.02 (10)
O4—C10—C8	112.11 (10)	N1—O2—C12	108.84 (9)
O4—C11—H11A	109.5	C10—O4—C11	115.35 (12)
O4—C11—H11B	109.5	C18—O5—C19	118.13 (14)
H11A—C11—H11B	109.5		
			
O1—C1—C2—C3	179.39 (14)	C10—C8—C12—O2	−91.49 (11)
C6—C1—C2—C3	1.0 (2)	C7—C8—C12—C13	−97.56 (11)
C1—C2—C3—C4	1.1 (3)	C9—C8—C12—C13	17.51 (15)
C2—C3—C4—C5	−1.8 (3)	C10—C8—C12—C13	148.16 (10)
C3—C4—C5—C6	0.3 (3)	O2—C12—C13—C14	−17.90 (17)
O1—C1—C6—C5	179.24 (12)	C8—C12—C13—C14	97.88 (15)
C2—C1—C6—C5	−2.5 (2)	O2—C12—C13—C18	162.62 (11)
O1—C1—C6—C7	−2.07 (18)	C8—C12—C13—C18	−81.60 (14)
C2—C1—C6—C7	176.20 (12)	C18—C13—C14—C15	0.5 (2)
C4—C5—C6—C1	1.8 (2)	C12—C13—C14—C15	−178.98 (14)
C4—C5—C6—C7	−176.77 (15)	C13—C14—C15—C16	−0.4 (3)
C1—C6—C7—N1	168.16 (12)	C14—C15—C16—C17	0.3 (3)
C5—C6—C7—N1	−13.2 (2)	C15—C16—C17—C18	−0.1 (3)
C1—C6—C7—C8	−8.22 (17)	C16—C17—C18—O5	179.75 (16)
C5—C6—C7—C8	170.40 (12)	C16—C17—C18—C13	0.2 (2)
N1—C7—C8—C9	−138.72 (11)	C14—C13—C18—O5	−179.97 (12)
C6—C7—C8—C9	38.09 (14)	C12—C13—C18—O5	−0.47 (18)
N1—C7—C8—C10	99.88 (12)	C14—C13—C18—C17	−0.4 (2)
C6—C7—C8—C10	−83.31 (13)	C12—C13—C18—C17	179.12 (13)
N1—C7—C8—C12	−14.94 (13)	C6—C7—N1—O2	−176.52 (11)
C6—C7—C8—C12	161.87 (10)	C8—C7—N1—O2	−0.02 (14)
C7—C8—C9—O1	−59.44 (13)	C2—C1—O1—C9	159.71 (12)
C10—C8—C9—O1	60.16 (13)	C6—C1—O1—C9	−21.95 (17)
C12—C8—C9—O1	−170.16 (10)	C8—C9—O1—C1	53.68 (14)
C7—C8—C10—O3	−23.21 (17)	C7—N1—O2—C12	16.69 (13)
C9—C8—C10—O3	−141.51 (13)	C13—C12—O2—N1	97.46 (11)
C12—C8—C10—O3	84.40 (15)	C8—C12—O2—N1	−25.18 (12)
C7—C8—C10—O4	158.97 (10)	O3—C10—O4—C11	−1.54 (19)
C9—C8—C10—O4	40.67 (14)	C8—C10—O4—C11	176.26 (11)
C12—C8—C10—O4	−93.43 (12)	C17—C18—O5—C19	−1.3 (2)
C7—C8—C12—O2	22.79 (11)	C13—C18—O5—C19	178.27 (14)
C9—C8—C12—O2	137.86 (11)		

### Hydrogen-bond geometry (Å, º)

**Table:**

*D*—H···*A*	*D*—H	H···*A*	*D*···*A*	*D*—H···*A*
C17—H17···O3^i^	0.93	2.42	3.3084 (19)	159

Symmetry code: (i) *x*+1, *y*, *z*.
